# Maßnahmen zur Hitze- und Starkregenvorsorge in Kitas und Pflegeeinrichtungen: Eine Evaluation von Risikowahrnehmung, Kommunikation und Informationsmaterialien

**DOI:** 10.1007/s00103-024-03876-8

**Published:** 2024-04-24

**Authors:** Anna Heidenreich, Lara-Heléne Deppermann, Annegret H. Thieken, Antje Otto

**Affiliations:** 1 Institut für Umweltwissenschaften und Geographie, Universität Potsdam, Karl-Liebknecht-Straße 24–25, 14476 Potsdam-Golm, Deutschland; 2 Weizenbaum-Institut, Hardenbergstraße 32, 10623 Berlin, Deutschland

**Keywords:** Klimaanpassung, Vulnerable Gruppen, Extremwetterereignisse, Risikokommunikation, Naturgefahren, Climate change adaptation, Vulnerable groups, Extreme weather events, Risk communication, Natural hazards

## Abstract

**Hintergrund und Ziel:**

Hitze und Starkregen können negative Auswirkungen auf die Gesundheit von Menschen auch in Deutschland haben. Insbesondere vulnerable Gruppen wie Kinder und Ältere sind einem erhöhten Risiko ausgesetzt und bedürfen der besonderen Vorsorge. Diese Arbeit untersucht, wie Gefahren durch Hitze und Starkregen in der kommunalen Verwaltung und bei Trägern von Kindertagesstätten und Pflegeeinrichtungen wahrgenommen werden und inwiefern hierzu ein Austausch zwischen kommunaler Ebene und Einrichtungen stattfindet. Eigens entwickelte Informationsmaterialien mit Handlungsempfehlungen zur Anpassung an Hitze und Starkregen, die sich an Einrichtungen richten, werden evaluiert.

**Methoden:**

Im Sommer 2021 fand eine quantitative Befragung von insgesamt 333 Teilnehmenden aus Stadtverwaltungen, Trägern und Einrichtungen (Kindertagesstätten und Pflegeeinrichtungen) statt. Zur statistischen Auswertung wurden deskriptive Analysen und Varianzanalysen durchgeführt.

**Ergebnisse:**

Die Risikowahrnehmung und auch das Handlungswissen bzgl. Hitze fielen höher als hinsichtlich Starkregen aus. Die Handlungsabsicht, Einrichtungen Unterstützung zur Anpassung aufzuzeigen, war bzgl. Hitze ebenfalls größer. Die Mehrheit der Befragten aus Stadtverwaltungen und von Trägern stand mit Einrichtungen über verschiedene Wege im Austausch und kommunizierte u. a. über die genannten Naturgefahren. Das Informationsmaterial wurde mehrheitlich positiv bewertet.

**Diskussion:**

Die Einrichtungen werden in Hinblick auf Hitze als stark betroffen angesehen. Die Sensibilisierung hinsichtlich Starkregens bedarf stärkerer Förderung. Die Rückmeldungen zu dem Informationsmaterial machen einen hohen Bedarf in diesem Bereich deutlich.

**Zusatzmaterial online:**

Zusätzliche Informationen sind in der Online-Version dieses Artikels (10.1007/s00103-024-03876-8) enthalten.

## Abstract (en)

**Background and aim:**

Heat and heavy rain can have negative health impacts for people in Germany. Vulnerable groups in particular, such as children and the elderly, are at increased risk and require special precautions. This paper examines how employers of the municipal administration and facilitating organisations perceive the risk of heat and heavy rain for daycare centres and care facilities, and to what extent an exchange takes place between the municipal level and the facilities. In addition, specially developed information materials with recommendations for action for adapting to heat and heavy rain that are aimed at such facilities were evaluated.

**Methods:**

In the summer of 2021, we conducted a quantitative survey. A total of 333 respondents from municipal administrations, facilitating organisations and institutions participated. Descriptive analyses and ANOVAs were conducted.

**Results:**

Risk perception and adaptation knowledge concerning heat was perceived higher than concerning heavy rain. The intention to support institutions in finding measures for adaptation was also higher with regard to heat. The majority of interviewees from municipal administrations and institutions communicated with institutions through various channels on different topics including the natural hazards mentioned. The information material was evaluated positively.

**Discussion:**

This article shows that facilities are seen as very affected by heat waves. Awareness towards heavy rainfall needs to be raised. The feedback on the information material clearly shows a high need in this area.

## Einleitung

Seit Beginn der Wetteraufzeichnungen in Deutschland im Jahr 1881 war die 10-Jahres-Periode 2013 bis 2022 die bisher wärmste Dekade [1]. Aktuellen Projektionen folgend werden Hitzewellen hierzulande zukünftig häufiger und intensiver auftreten [2]. Insbesondere ältere Menschen, Schwangere, Säuglinge, (Klein‑)Kinder, Menschen mit Erkrankungen und Menschen, die sich viel im Freien aufhalten (müssen), sind gegenüber Hitze vulnerabel [3–6]. Die gesundheitlichen Belastungen führen dabei – v. a. in der Gruppe der hochbetagten Personen – zu einer erhöhten Mortalität [7, 8].

Neben Hitzetagen wird in Deutschland eine Zunahme der Anzahl von Starkregentagen angenommen [2]. Bereits in den letzten Jahren ereigneten sich diverse Starkregenereignisse, die zu Überflutungen und Schäden an Gebäuden und Infrastruktur führten [9]. In extremeren Fällen können lebensbedrohliche Situationen entstehen, u. a. für Menschen, die auf die Unterstützung anderer angewiesen sind, um Warnungen zu erhalten und zu verstehen, Anpassungsmaßnahmen zu treffen und/oder sich in Sicherheit zu bringen. Dies trifft z. B. auf ältere und pflegebedürftige Menschen sowie Kinder zu. Infolge der Überflutungen 2021 in Rheinland-Pfalz und Nordrhein-Westfalen entfielen über zwei Drittel der Todesfälle auf Menschen über 60 Jahre [10] und in einer Pflegeeinrichtung verstarben 12 Menschen [11].

Vor diesem Hintergrund erweist sich die gesundheitliche Anpassung an Wetterextreme als notwendig für eine resiliente Gesellschaft [12]. Einrichtungen wie Kindertagesstätten (Kitas) und Pflegeeinrichtungen, in denen vulnerable Personen (zeitweise) betreut werden, stehen in der Verantwortung, sich auf Hitze und Starkregen vorzubereiten und während derartiger Ereignisse Schutzmaßnahmen durchzuführen. Es gibt zahlreiche Empfehlungen für Anpassungsmaßnahmen (kurz: Handlungsempfehlungen), welche von Einrichtungen durchgeführt werden können. Diese unterscheiden sich u. a. nach technischen, organisatorischen und pflegerischen Aspekten [13] sowie dem Zeithorizont der Umsetzung (kurz-, mittel-, langfristig). Kommunale Verwaltungen gelten hierbei mitunter als zuständig dafür, die Gefährdung von Einrichtungen zu erfassen und sie bzw. deren Träger zu sensibilisieren und zu informieren [3, 14, 15]. Jedoch zeigen u. a. Befragungsergebnisse, dass Kommunen bislang nur bedingt auf diese Herausforderung vorbereitet und häufig nicht ausreichend ausgestattet sind [16, 17]. Zudem ist bislang nur wenig darüber bekannt, wie Hitze und Starkregen in der kommunalen Verwaltung und bei Trägern von Kitas und Pflegeeinrichtungen wahrgenommen werden, inwiefern hierzu ein Austausch zwischen kommunaler Ebene und Einrichtungen stattfindet und wie Informationsmaterialien eingeschätzt werden. Dieser Artikel zielt darauf ab, die Erkenntnisse in diesem Bereich zu erweitern.

### Hitze und Starkregen in Kitas und Pflegeeinrichtungen

Studien zu Kitas aus Schweden und Deutschland deuten mitunter auf eine hohe Hitzebelastung und auf die Umsetzung diverser Vorsorge- und Schutzmaßnahmen hin, wobei festgestellt wird, dass es an einem systematischen Vorgehen in der Hitzevorsorge, an ausreichender Integration dieser Themen in die Ausbildung und an kinderspezifischen Handlungsempfehlungen mangelt [18–21]. Die Gebäude und Außenbereiche erweisen sich zudem häufig als nicht ausreichend an Hitze angepasst, da Begrünung und Beschattung fehlen [22].

Untersuchungen, die sich mit dem Umgang mit Hitze in stationären Pflegeeinrichtungen [23–25] und ambulanter Pflege [26] in Deutschland beschäftigen, zeigen ebenfalls, dass diverse Maßnahmen zur Hitzevorsorge ergriffen werden. Die Wahrnehmung und das Wissen über Hitzerisiken sowie vorausschauendes Handeln werden jedoch angesichts der Gefährdung, die Hitze für diese Altersgruppe darstellt, als zu gering bewertet [3, 23, 26]. Zudem können neben den betreuten Kindern und gepflegten Personen auch die Mitarbeitenden selbst aufgrund der z. T. körperlich schweren Arbeit und der bei Hitze mitunter steigenden Arbeitsbelastung gesundheitlich von Hitze beeinträchtigt sein [13, 18, 25].

Krisen- und Katastrophenfälle in Kitas und Pflegeeinrichtungen, die durch Starkregenereignisse ausgelöst werden, spielen in der auf Deutschland bezogenen Literatur bisher kaum eine Rolle [27], obgleich diese Einrichtungen in der Vergangenheit von Überflutungen betroffen waren [28]. Praxisempfehlungen für diese Einrichtungen beschränken sich zumeist auf Stromausfälle und Evakuierungen bzw. Räumungen aufgrund von Bränden [29, 30]. Studien zeigen, dass zwei Drittel der Pflegeeinrichtungen nicht über Notfallpläne für Extremwetterereignisse verfügen [31], dass Pfleger:innen ein eher geringes Risikobewusstsein aufweisen [32] und dass es sehr große Unterschiede dahingehend gibt, wie gut sich Pfleger:innen auf Krisensituationen vorbereitet fühlen [33]. Ein Vergleich der Erfahrungen aus 3 Pflegeeinrichtungen während des Elbehochwassers 2013 zeigte, dass die Vorlaufzeit und die Kommunikation mit den Einsatzorganisationen für ein gutes Gelingen der Räumung ausschlaggebend sind [34]. In der ambulanten Pflege sind Evakuierungen und Räumungen u. a. aufgrund der Dezentralität der Pflegebedürftigen besonders herausfordernd und die Versorgung bei Extremwetterereignissen ist durch Verkehrsbehinderungen eingeschränkt [27, 32].

Für die systematische Vorsorge und Umsetzung angemessener Maßnahmen in Kitas, Pflegeeinrichtungen und in der ambulanten Pflege stellen fehlende Personalressourcen eine große Herausforderung dar [18, 34, 35]. Denn diese sind neben Wissen und finanziellen Mitteln für die Umsetzung bestimmter Anpassungsmaßnahmen notwendig. Eine eigene Auswertung bundesweit bewilligter Fördermittel (Stand: 01.01.2023; [36]) zeigt, dass bei Kitas und Pflegeeinrichtungen vor allem konkrete Maßnahmen gegen Hitze gefördert werden – allen voran Verdunkelungsmaßnahmen und Schutzverglasungen an Fenstern, Maßnahmen der Verschattung, Begrünung und Entsiegelung im Außenbereich sowie seltener die Bereitstellung von Wasserspendern und die Gebäudedämmung.

Zur Stärkung der Risikowahrnehmung, des Risikobewusstseins und des Handlungswissens werden informative und praxisnahe Informationsmaterialien wie Leitfäden, Checklisten, Schulungen und Musternotfallpläne empfohlen [31, 34, 37, 38]. Dem wurde in den letzten Jahren für das Themengebiet Hitze entsprochen und diverse Informationsmaterialien, Schulungen u. Ä. für Pflegeeinrichtungen und ambulante Pflege sowie zunehmend auch für Kitas angeboten [39]. Dies mag zu einem zunehmend unübersichtlichen Angebot verschiedener nebeneinander existierender Materialien führen, deren Qualität bislang nicht (vergleichend) evaluiert wurde. Für die Starkregenvorsorge gibt es bislang noch kaum vergleichbare Angebote (für einen Überblick zu relevanten Angeboten für Kitas und Pflegeeinrichtungen zur Hitze- und Starkregenvorsorge siehe [39]).

### Iterativer Co-Design-Erstellungsprozess von Kommunikationsmaterialen für Kitas und Pflegeeinrichtungen

Ob Kommunikationsformate tatsächlich zur Eigenvorsorge motivieren, ist von verschiedenen individuellen Faktoren abhängig, u. a. von der Betroffenheit, der Risikowahrnehmung, der Überzeugung von der Wirksamkeit und Umsetzbarkeit von Maßnahmen und der wahrgenommenen persönlichen Verantwortung [40, 41]. Neben diesen psychologischen Einflussfaktoren sind die Art und Gestaltung der Risikokommunikation relevant für die Motivation zur Eigenvorsorge, wobei bislang kaum Erkenntnisse aus Wirkevaluationen vorliegen [40, 42–44].

Bei Informationsmaterialien und -veranstaltungen wird es als wichtig erachtet, dass die Zielgruppen bereits in der Erstellungsphase aktiv in einen Co-Designprozess einbezogen werden [41, 45, 46], bspw. durch Interviews, Workshops oder Befragungen. Dies dient dazu, möglichst bedarfsgerechte, verständliche, ansprechend gestaltete und an die Nutzenden angepasste Materialien zu erarbeiten [41, 45, 46].

**Abb. 1:**
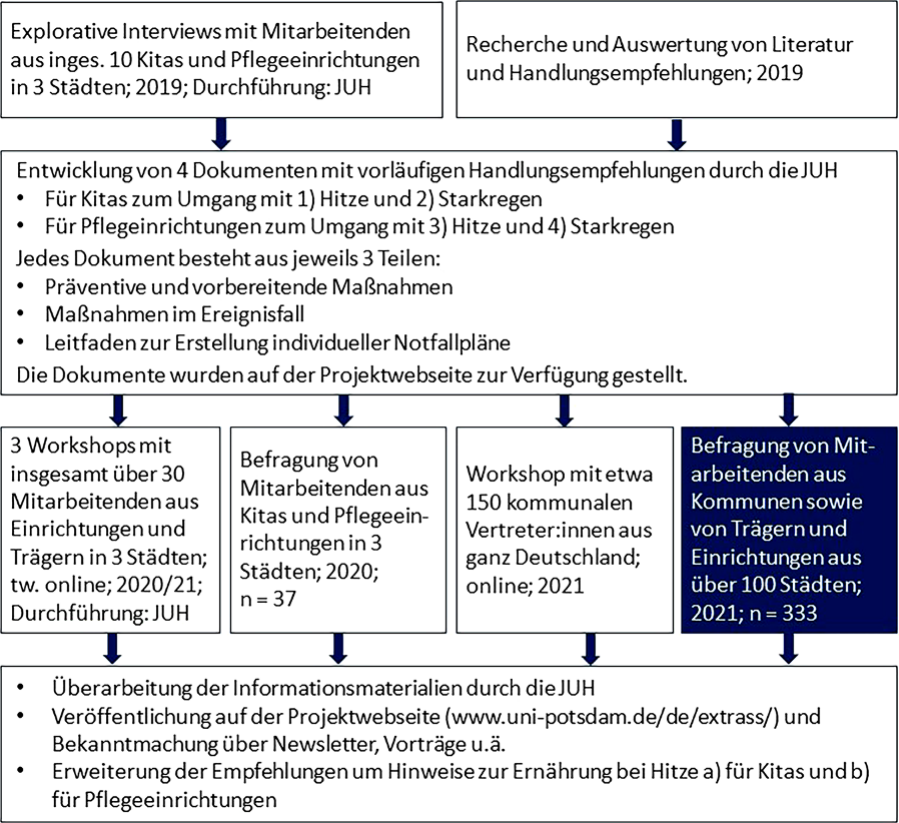
Iterativer Prozess zur Erstellung von Informationsmaterialien mit Handlungsempfehlungen für Kitas und Pflegeeinrichtungen zu Hitzewellen und Starkregen. (Eigene Abbildung. *JUH* Johanniter-Unfall-Hilfe e. V.)

Diese Hinweise berücksichtigend startete 2018 ein iterativer Prozess, in dem Mitarbeitende der Johanniter-Unfall-Hilfe e. V. (JUH) mit Unterstützung der Autorinnen Informationsmaterialien mit Handlungsempfehlungen für Kitas und Pflegeeinrichtungen zu den Themen Hitze und Starkregen erstellten [47, 48]. In diesem Prozess wurden die Zielgruppen (Kitas, stationäre Pflegeeinrichtungen) sowie mögliche Multiplikatoren (kommunale Verwaltungen und Trägerinstitutionen) wiederholend und in unterschiedlichen Formaten beteiligt und Pretests mit den Informationsmaterialien durchgeführt (Abb. 1).

Die ersten Versionen von 4 Dokumenten (1. Kitas/Hitze, 2. Kitas/Starkregen, 3. Pflegeeinrichtungen/Hitze und 4. Pflegeeinrichtungen/Starkregen) basierten auf den Erkenntnissen aus explorativen Interviews und der Auswertung vorhandener Literatur. Die entwickelten Informationsmaterialien beinhalten Empfehlungen zu präventiven und vorbereitenden Maßnahmen, Maßnahmen im Ereignisfall und einen Leitfaden zur Erstellung individueller Notfallpläne. Sie zielen darauf ab, in Kitas und Pflegeeinrichtungen für Extremwetterereignisse zu sensibilisieren, zu informieren und zur Umsetzung von Anpassungsmaßnahmen anzuregen. Damit eignen sie sich u. a. dazu, von Multiplikatoren wie kommunalen Verwaltungen und Trägerinstitutionen an soziale Einrichtungen weitergegeben zu werden.

Nach der Erstellung der vorläufigen Materialien wurden in 3 Städten Workshops und Befragungen mit Vertreter:innen der kommunalen Verwaltungen, sozialer Einrichtungen und deren Trägern durchgeführt. Zum einen wurden hierbei weitere Informationen gesammelt, u. a. hinsichtlich des Austauschs zwischen den unterschiedlichen Zielgruppen und zu bisherigen Erfahrungen mit Hitze und Starkregen. Zum anderen wurden die Informationsmaterialien vorgestellt und von den Teilnehmenden bewertet, kommentiert und diskutiert. Die Anmerkungen sind in eine überarbeitete Fassung der Materialien eingegangen.

Dieser Artikel stellt die Ergebnisse einer breiten Befragung von Mitarbeitenden in Kommunalverwaltungen, Einrichtungen und Trägerinstitutionen dar und geht den folgenden Forschungsfragen nach:

1. Wie nehmen die verschiedenen Akteure die Betroffenheit von Hitze und Starkregen sowie Möglichkeiten zur Anpassung wahr?
2. Inwiefern besteht zwischen Stadtverwaltungen und Trägern einerseits und Kitas und Pflegeeinrichtungen andererseits eine Kommunikation und gibt es einen Austausch zu den Themen Hitze und Starkregen?
3. Wie werden die entwickelten Informationsmaterialien von den verschiedenen Akteuren bewertet?

## Methoden

### Durchführung und Auswertung

Zur Beantwortung der genannten Fragestellungen wurde im Sommer 2021 eine deutschlandweite Befragung durchgeführt. Es wurden Einladungen zur Befragungsteilnahme an 1807 vorab online recherchierte E‑Mail-Adressen von Stadtverwaltungen (1232) und Trägern (575) verschickt (Tab. A1 im Onlinematerial). Adressiert waren Mitarbeitende der Stadtverwaltungen aller großen und kreisfreien mittelgroßen Städte Deutschlands (insgesamt 104 Städte, siehe [49]), die sich mit umwelt-, sozial- oder gesundheitsrelevanten Themen befassen, sowie Mitarbeitende bei Trägern sozialer Einrichtungen. Einige Empfänger:innen leiteten die E‑Mail weiter, somit wurden vermutlich auch Mitarbeitende von Verwaltungen und Trägern anderer Kommunen und Verwaltungsebenen erreicht. Zusätzlich wurde auf der Projektwebseite über die Befragung informiert und zur Teilnahme eingeladen. Im Folgenden werden die Teilnehmenden der beiden adressierten Gruppen „Stadtverwaltungen“ und „Träger“ genannt.

Für die statistische Auswertungen wurde Microsoft Excel (Redmond, WA, USA) und IBM SPSS Statistics, Version 28 (Armonk, NY, USA) zur Durchführung deskriptiver Analysen und Varianzanalysen genutzt. Im Ergebnisteil werden deskriptive Ergebnisse berichtet, während die zusätzlichen Onlinematerialien (Tab. A1–A9) weitere Informationen zur Stichprobe und zu Gruppenunterschieden (Varianzanalysen) bieten.

### Fragebogen

Der Fragebogen bestand aus 17 geschlossenen und 4 offenen Fragen zur Kommunikation mit Einrichtungen über Hitze und Starkregen, zur Einschätzung der Risikowahrnehmung dieser Extremwetterereignisse, zu der Bewertung der Informationsmaterialien und der Absicht einer Weiterleitung dieser.

Zur Erfassung der Risikowahrnehmung wurden allen Befragten je 3 Aussagen zu beiden Naturgefahren – Hitze und Starkregen – mit der Bitte präsentiert, diese jeweils auf einer Skala von 1 = „ich stimme gar nicht zu“ bis 6 = „ich stimme vollkommen zu“ zu bewerten. Hierbei ging es um die Einschätzung der Betroffenheit der Einrichtungen gegenüber Hitze und Starkregen („Die Einrichtungen unserer Stadt/unseres Trägers sind sehr stark von Hitzewellen/Starkregen betroffen“), der Thematisierung von Hitze und Starkregen in den Einrichtungen („In den Einrichtungen unserer Stadt/unseres Trägers stand der Umgang mit Hitze/Starkregen schon oft auf der Tagesordnung“) und deren Handlungswissen („Im Falle einer Hitzewelle/von Starkregen wüssten Mitarbeitende in den Einrichtungen unserer Stadt/unseres Trägers, welche Schutzmaßnahmen sie ergreifen können“).

Um die wahrgenommene Dringlichkeit der beiden Naturgefahren zu erfassen, wurde die Handlungsabsicht hinsichtlich der Umsetzung von Anpassungs- oder Vorsorgemaßnahmen erfragt. Die Befragten der Träger und Stadtverwaltungen wurden nach ihren Absichten gefragt, den Einrichtungen Unterstützungsmöglichkeiten zur Durchführung von Anpassungsmaßnahmen aufzuzeigen sowie ihnen die Durchführung konkreter Schutzmaßnahmen zu empfehlen.

Weitere Fragen bezogen sich auf den Kontakt zwischen Stadtverwaltungen und Kitas bzw. Einrichtungen, die genutzten Kommunikationskanäle und kommunizierten Inhalte. Darüber hinaus konnten die Befragten die genannten Informationsmaterialien mittels eines Polaritätsprofils bewerten, angeben, ob sie diese weitergeben würden, und in einem offenen Feld weitere Rückmeldungen eingeben.

### Stichprobenbeschreibung

Vom 09.06.2021 bis zum 31.07.2021 schlossen insgesamt 333 Teilnehmende die Befragung ab. Darunter waren 198 Angestellte von Stadtverwaltungen und 100 Mitarbeitende von Trägern. Zusätzlich nahmen 35 Mitarbeitende aus Pflege- und Kindertageseinrichtungen teil. Diese 35 Teilnehmenden gehörten nicht zur ursprünglich angesprochenen Zielgruppe; die Angaben zu einigen Fragen wurden aber mit ausgewertet, um die Perspektive der direkt Betroffenen in dieser Analyse nicht unbeachtet zu lassen.

Die meisten Befragten aus den Stadtverwaltungen kamen aus Bayern, Thüringen und Nordrhein-Westfalen. Bei den Trägereinrichtungen waren die Bundesländer Rheinland-Pfalz und Niedersachsen mit je über 20 % am stärksten vertreten. In Mecklenburg-Vorpommern nahmen keine Mitarbeitenden der Kommunen und in Thüringen keine Mitarbeitenden der Träger an der Befragung teil, ansonsten waren alle Bundesländer repräsentiert. 76,8 % der Teilnehmenden aus Stadtverwaltungen gaben an, in Gesundheits- und Sozialabteilungen tätig zu sein. 8,1 % befassen sich bei ihrer Arbeit mit Umwelt- und Klimathemen und 13,6 % gaben einen anderen Tätigkeitsschwerpunkt an.

## Ergebnisse

**Abb. 2:**
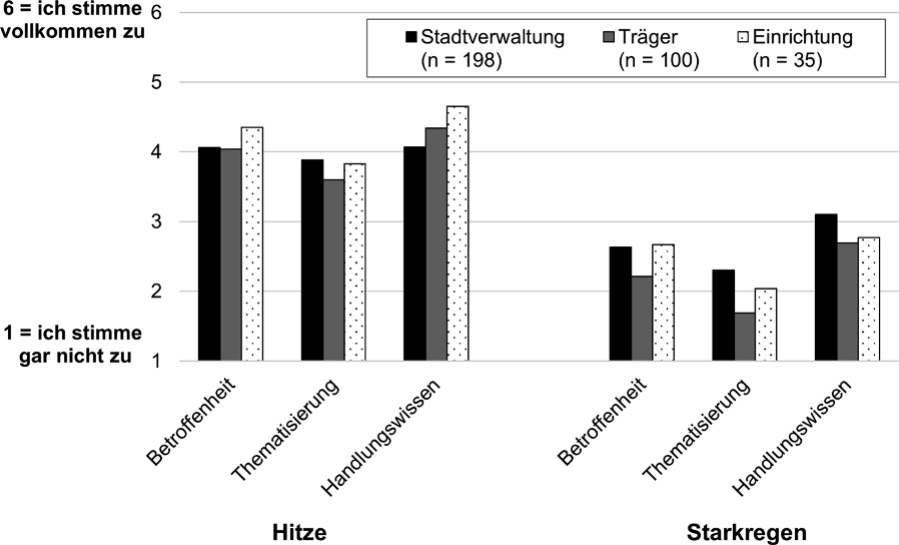
Wahrnehmung der Themen Hitze und Starkregen. Verzeichnet ist das arithmetische Mittel je Teilnehmergruppe. Wortlaut der Frageformulierungen siehe Abschn. „Fragebogen“. (Eigene Abbildung)

Es zeigte sich im Durchschnitt eine geringe bis mittelhohe Zustimmung bei allen 3 Fragen zur Risikowahrnehmung (Abb. 2). Alle Gruppen nahmen eine höhere Betroffenheit, Thematisierung und ein höheres Handlungswissen hinsichtlich Hitze wahr. Die Unterschiede zwischen den beiden Naturgefahren sind signifikant, die befragten Gruppen unterscheiden sich hingegen nicht signifikant (zweifaktorielle Varianzanalysen in Tab. A4).

**Abb. 3:**
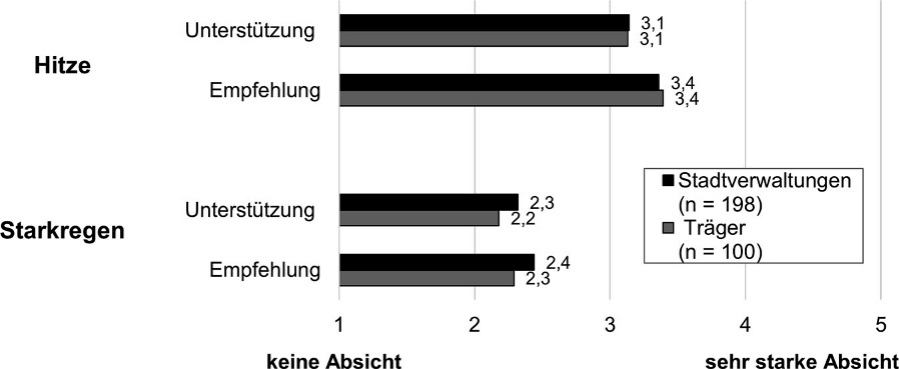
Handlungsabsicht, in den kommenden Wochen Einrichtungen Unterstützungsmöglichkeiten zur Durchführung von Schutzmaßnahmen gegen Hitzewellen/Starkregen aufzuzeigen und Einrichtungen die Durchführung einer oder mehrerer der vorgestellten Schutzmaßnahmen gegen Hitzewellen/Starkregen zu empfehlen. Verzeichnet ist das arithmetische Mittel für die Teilnehmergruppen Stadtverwaltungen und Träger. Wortlaut der Frageformulierungen siehe Tab. A5 in den Onlinematerialien. (Eigene Abbildung)

Insgesamt bewegten sich die Handlungsabsichten hinsichtlich der Umsetzung von Anpassungs- oder Vorsorgemaßnahmen ungefähr um den Skalenmittelwert von 3 (von 1 = „keine Absicht“ bis 5 = „sehr starke Absicht“) oder lagen darunter. Wie Abb. 3 und zweifaktorielle Varianzanalysen (Tab. A5) zeigen, waren die Handlungsabsichten der Stadtverwaltungen und Träger, in den kommenden Wochen Einrichtungen Unterstützungsmöglichkeiten zur Durchführung von Schutzmaßnahmen aufzuzeigen, bei Hitze stärker als bei Starkregen. Ebenso war die Absicht, Einrichtungen die Durchführung einer oder mehrerer der vorgestellten Schutzmaßnahmen zu empfehlen, bezogen auf Hitze stärker als bezogen auf Starkregen.

42,0 % der Teilnehmenden der Träger (T) und 33,8 % der Gruppe der Stadtverwaltungen (S) gaben an, dass es ihres Wissens eigene Materialien zum Umgang mit Hitze und/oder Starkregen gibt (Tab. A6). Wenn eigene Materialien vorhanden waren, gab es häufiger Materialien zum Umgang mit Hitze (T: 41,0 %; S: 31,3 %) als zum Umgang mit Starkregen (T: 6,0 %; S: 12,6 %). Einige gaben an, Materialien zu beiden Themenbereichen zu haben (T: 5,0 %; S: 20,0 %). Inwiefern diese Materialien speziell auf bestimmte Einrichtungen zugeschnitten sind, wurde nicht erhoben.

Rund 3 Viertel der Befragten aus der Stichprobe der Stadtverwaltungen äußerten, dass sie grundsätzlich mit Einrichtungen im Austausch stehen: Mit Kitas standen 47,0 % in Kontakt, mit Pflegeeinrichtungen 44,9 % und 15,7 % gaben beide Gruppen an. Unter den Befragten der Träger standen 88,0 % grundsätzlich mit Einrichtungen im Austausch: Die Mehrheit nannte Kitas (63,0 %), etwa halb so viele (31,0 %) Pflegeeinrichtungen und nur vereinzelt beide (6,0 %; Tab. A7).

**Abb. 4:**
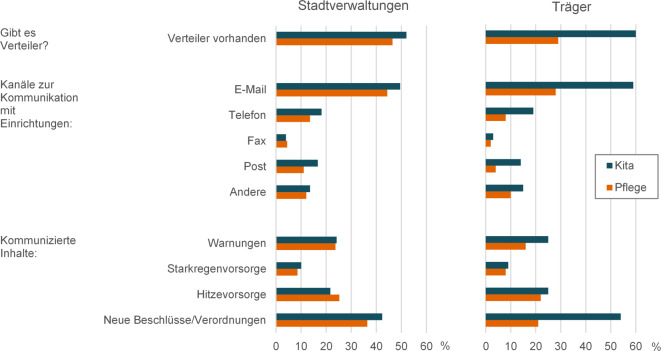
Verteiler und Kommunikationskanäle von Stadtverwaltungen (*n* = 198) und Trägern (*n* = 100), über die Informationen an Kitas und Pflegeeinrichtungen verteilt werden, sowie kommunizierte Inhalte (jeweils Mehrfachauswahl möglich, relativer Anteil in Prozent). Ausführliche Angaben siehe Tab. A8 im Onlinematerial. (Eigene Abbildung)

Gefragt, ob in ihren Abteilungen/Arbeitsgruppen Verteiler zur gezielten Ansprache von Kitas und/oder Pflegeeinrichtungen vorhanden seien, nannte etwa die Hälfte der Befragten aus Stadtverwaltungen Verteiler für Kitas und etwas seltener für Pflege. Insgesamt gab ein Fünftel Verteiler für beide Einrichtungsarten an (Mehrfachantworten möglich). Unter den Befragten der Träger nannten 60,0 % Verteiler mit Kitas, 29,0 % mit Pflegeeinrichtungen und 11,0 % beide Gruppen. Ein Großteil der Kommunikation verläuft über E‑Mail, seltener über Telefon oder Post, per Fax nur vereinzelt (Abb. 4). Als weitere Kommunikationskanäle wurden hauptsächlich persönliche Treffen (z. B. bei Arbeitskreisen, Abteilungskonferenzen oder Besprechungen) sowie vereinzelt Homepage, Messenger, Apps und das Intranet genannt. Viele Befragte berichteten, neue Beschlüsse oder Verordnungen zu kommunizieren. Etwa ein Viertel beider befragten Gruppen gab zudem konkrete Warnungen weiter, wobei möglich ist, dass einige sich auch auf Warnungen vor anderen Gefahren als Hitze und Starkregen bezogen. Informationen zur Hitzevorsorge wurden etwa doppelt so häufig genannt wie solche zur Starkregenvorsorge (Abb. 4; Tab. A8).

**Abb. 5:**
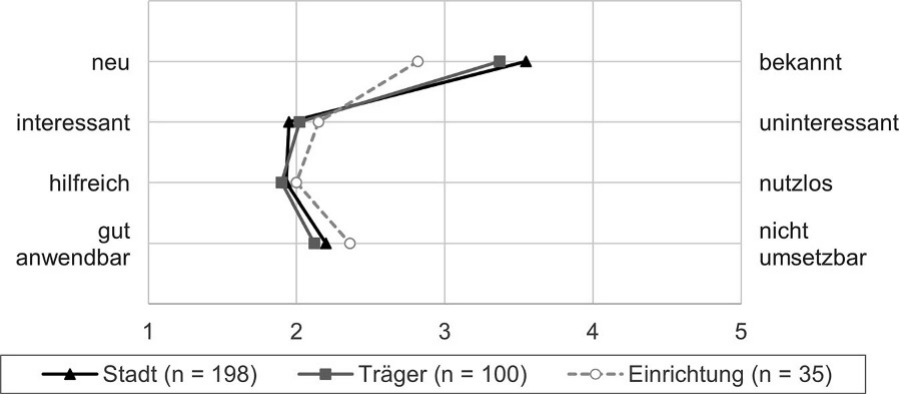
Polaritätsprofil der gemittelten Antwortoptionen auf die Frage: „Insgesamt erscheinen mir die Handlungsempfehlungen für Kindertagesstätten und/oder Pflegeeinrichtungen …“ Die Mittelwerte (*M*) und Standardabweichungen (*SD*) der einzelnen Gruppen sind in Tab. A9 im Onlinematerial dargestellt. (Eigene Abbildung)

Zur Bewertung der erarbeiteten und verschickten Informationsmaterialien wurden Fragen in Form eines Polaritätsprofils genutzt (Abb. 5). Das bedeutet, dass die Befragten auf die vorangestellte Aussage: „Insgesamt erscheinen mir die Handlungsempfehlungen für Kindertagesstätten und/oder Pflegeeinrichtungen …“, angeben konnten, wie sie ihre Einschätzung auf einem Kontinuum zwischen den Polen neu – bekannt, interessant – uninteressant, hilfreich – nutzlos, gut anwendbar – nicht umsetzbar jeweils verorten würden. Die Handlungsempfehlungen wurden als eher interessant, hilfreich und gut anwendbar bewertet. Hinsichtlich der Neuheit/Bekanntheit der Handlungsempfehlungen wird sichtbar (Abb. 5), dass sich die Antworten dichter um das Skalenmittel bewegen. Befragte aus Stadtverwaltungen und von Trägern bewerten die Handlungsempfehlungen deskriptiv eher als bekannt als jene aus Einrichtungen, wobei dieser Gruppenunterschied nicht signifikant ist, wie einfaktorielle Varianzanalysen zeigen (Tab. A9).

**Abb. 6:**
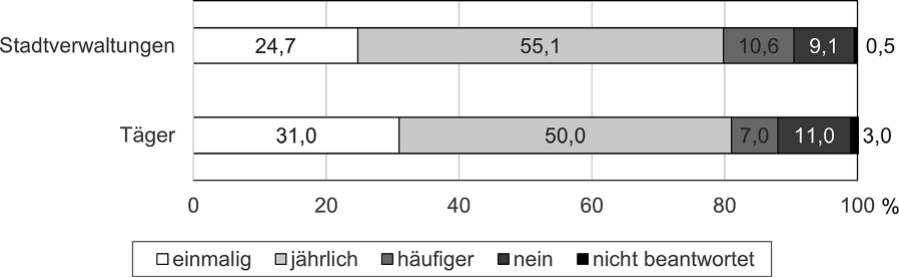
Anteil der Befragten, die die Handlungsempfehlungen weiterleiten würden, und Häufigkeit der Weiterleitung. *n* = 198 Mitarbeitende aus Stadtverwaltungen und *n* = 100 Mitarbeitende von Trägern. (Eigene Abbildung)

Die Mehrheit der Befragten gab an, dass sie die Materialien an Einrichtungen weitergeben würden (S: 90,4 %; T: 88,0 %, Abb. 6). Etwa die Hälfte der Befragten würde dies einmal im Jahr tun, einige würden sie sogar häufiger weiterleiten. Ungefähr ein Viertel bzw. ein Drittel beabsichtigte die einmalige Weiterleitung. Nur etwa ein Zehntel der Befragten gab an, die Materialien nicht an Einrichtungen weiterleiten zu wollen. Als Gründe dafür wurden eigene vorhandene Materialien zur Thematik und vereinzelt die Bekanntheit der Handlungsempfehlungen oder die bezweifelte Umsetzbarkeit insbesondere aus mangelnder Finanzierbarkeit genannt.

## Diskussion

Dieser Artikel liefert Erkenntnisse auf der Grundlage von Befragungsdaten zur Hitze- und Starkregenvorsorge in sozialen Einrichtungen, zur Kommunikation zwischen Stadtverwaltungen und Einrichtungen sowie zur Bewertung eines ausgewählten Informationsmaterials. Die Befragungsergebnisse deuten darauf hin, dass die Befragten die Einrichtungen als sehr betroffen hinsichtlich Hitze einschätzen, und ihnen wird von Trägern und Stadtverwaltungen ein recht hohes Risikobewusstsein und Handlungswissen zugeschrieben. Die Ergebnisse decken sich hinsichtlich der Betroffenheit mit der vorhandenen Literatur, die auf den Umgang mit Hitze in diesen Einrichtungen eingeht [18, 19, 22, 23]. Unabhängig davon, wie gut die Einrichtungen Bescheid wüssten, was im Falle von Hitze oder Starkregen zu tun sei (Handlungswissen, Abb. 2), wurde im Rahmen der Befragung von einem Mangel an strukturierter Vorsorge berichtet. Nach Aussage vieler Befragter wird die Umsetzung u. a. durch mangelhafte personelle und finanzielle Ressourcen erschwert. Dies deckt sich mit Erkenntnissen aus der Literatur [18, 34, 35].

Die verschiedenen befragten Zielgruppen – Mitarbeitende bei Trägern und Stadtverwaltungen – unterschieden sich im Hinblick auf die Risikowahrnehmung und Einschätzung der Einrichtungen kaum. Unterschiede werden jedoch in der Wahrnehmung beider Naturgefahren deutlich: Beim Thema Starkregen werden sowohl die Betroffenheit als auch das Bewusstsein und Wissen der Einrichtungen deutlich niedriger als hinsichtlich Hitze eingeschätzt. Dies korrespondiert mit einer mangelnden Studienlage zu diesem Thema. Das Thema der Starkregengefährdung für soziale Einrichtungen und mögliche Vorsorge- und Schutzmaßnahmen sollte in den Einrichtungen selbst, aufseiten der kommunalen Verwaltungen und Träger, aber auch in der Wissenschaft daher stärker auf die Agenda gesetzt werden. Die Dringlichkeit wurde im Zuge des Hochwassers 2021, das auch Todesfälle in sozialen Einrichtungen verursachte, erneut deutlich. Derartige Ereignisse können das Risikobewusstsein und den Handlungsdruck für die Umsetzung von Anpassungsmaßnahmen steigern [50], allerdings haben über 95 % der Befragten vor Einsetzen des Hochwassers die Befragung abgeschlossen, sodass hierzu keine Aussagen getroffen werden können.

Die Absicht, in der nahen Zukunft Anpassungsmaßnahmen zu empfehlen oder Einrichtungen Möglichkeiten zur Umsetzung aufzuzeigen, war seitens der Befragten aus Stadtverwaltungen und Trägern gering bis mittelhoch ausgeprägt. Dies kann darauf zurückzuführen sein, dass konkrete Maßnahmen Ressourcen sowie eine gute Übersicht über die Lage in den Einrichtungen hinsichtlich Hitze und Starkregens benötigen. Eine Möglichkeit, mehr über die individuelle Exposition gegenüber Naturgefahren zu erfahren, stellen Gefahrenkarten dar. Diese sollten daher aktiv an Einrichtungen kommuniziert werden. Hinsichtlich der Handlungsabsicht zeigt sich, dass die Zustimmungswerte für Hitze größer ausfallen als für Starkregen. Welche psychologischen und anderweitigen Faktoren dem zugrunde liegen, müssen nachfolgende Studien klären. Gegebenenfalls wird Hitze eine höhere Dringlichkeit beigemessen oder aber die Umsetzbarkeit und Nützlichkeit von Anpassungsmaßnahmen sowie die eigene Verantwortlichkeit für deren Durchführung werden bei Hitze höher bewertet als bei Starkregen.

Die Mehrheit der Befragten von Stadtverwaltungen und Trägern gab an, mit Einrichtungen (häufiger mit Kitas als mit Pflegeeinrichtungen) in Kontakt zu stehen. Den Einrichtungen werden dabei vereinzelt Informationen zur Starkregen- und etwa doppelt so häufig solche zur Hitzevorsorge mitgeteilt. Vermehrt wurde angegeben, dass auch Warnungen kommuniziert werden, eine Angabe, die sich in anderen Befragungen nicht bestätigen lässt (z. B. [9]). Die Weitergabe an Informationen und Warnungen sollte vor dem Hintergrund der hohen Vulnerabilität der betreuten Personen und der ggf. steigenden Arbeitsbelastung der Mitarbeitenden bei Naturgefahren in den Einrichtungen verstärkt werden. Beispielsweise im Rahmen der Erarbeitung von kommunalen Hitzeaktionsplänen sollten die Verantwortlichkeiten und Abläufe hierzu festgelegt werden.

Die Informationsmaterialien wurden von den Befragten insgesamt sehr positiv bewertet. Aus den Rückmeldungen zeigt sich ein Bedarf für derartige Materialien. Anmerkungen zu konkreten Punkten und einzelnen Ungenauigkeiten wurden eingearbeitet und aktualisierte Versionen veröffentlicht. Zudem wurde dem Bedarf nach Ernährungsempfehlungen entsprochen (Abb. 1).

Der iterative Prozess war erkenntnisbringend für die Erstellung der Informationsmaterialien. Ausgehend von dieser Studie sind eine kooperative Erstellung und Evaluierung derartiger Materialien gemeinsam mit Praxispartnern zu empfehlen, wie dies auch der Hitzeschutzplan des Bundesministeriums für Gesundheit vom Juni 2023 vorschlägt [51]. Für die Erstellung von Hitzeaktionsplänen sollte der Kontakt zu Einrichtungen für vulnerable Gruppen ebenfalls zentraler Bestandteil sein. Zudem ist es ratsam, in kommenden Untersuchungen verschiedene Materialien vergleichend zu evaluieren. Handelt es sich um Materialien, welche in konkreten Projekten erstellt wurden, ist unbedingt sicherzustellen, wie diese nach Abschluss der Projekte aktuell gehalten und beworben werden können.

Bei der Interpretation der Ergebnisse ist zu beachten, dass die Daten über eine Onlinebefragung erhoben wurden, die trotz einer sorgfältigen Recherche nach Kontakten unter Trägern und Verwaltungen möglicherweise nicht alle erreichen konnte. Darüber hinaus spiegeln die Ergebnisse aufgrund der Städteauswahl vor allem die Erfahrungen und Einschätzungen aus großen und mittelgroßen Städten wider. Ob es in kleinen Kommunen abweichende Ergebnisse gibt, könnte in zukünftigen Forschungsarbeiten untersucht werden.

Zudem ist eine Verzerrung der Stichprobe („self-selection bias“) wahrscheinlich, da Personen, die bereits ein Interesse an der Thematik Hitze, Starkregen oder Klimawandelanpassung im Allgemeinen haben, vermutlich eher teilnahmen. An der Befragung nahmen deutlich mehr Mitarbeitende der Stadtverwaltung, die im Bereich Gesundheit/Soziales tätig sind, teil. Sie sahen sich offenbar deutlich häufiger zuständig für die Thematik als Kolleg:innen der Bereiche Klima/Umwelt, die ebenfalls adressiert waren. Eine enge Zusammenarbeit zwischen den verschiedenen Fachbereichen und weiteren Akteuren ist allerdings außerordentlich wichtig, um einen Schutz der Bevölkerung zu gewährleisten.

Diese Studie zeigt einen Bedarf für konkret an Einrichtungen adressierte Informationen zum Umgang mit Hitzebelastungen und Starkregen auf. Diese sollten für Einrichtungen leicht zugänglich sein oder sogar zugeschickt werden und aktuell gehalten werden. Eine reine Bereitstellung reicht jedoch nicht aus, auch eine Unterstützung der Einrichtungen und ein Austausch über Möglichkeiten der Umsetzung sind notwendig. Daneben gibt es Hinweise [24], dass Überprüfungen der Umsetzung von Schutzmaßnahmen im Eintrittsfall bei Hitze den Vorteil haben, dass diese Aufgabe – neben vielen anderen Anforderungen an das Personal – nicht so schnell aus dem Blick gerät. Insgesamt bedarf es einer Sensibilisierung bezüglich Starkregens, wie die Ereignisse im Juli 2021 verdeutlichen. Aber auch die Hitzebelastungen, bei denen die Sensibilisierung bereits ausgeprägter ist, müssen aufgrund der jährlich wiederkehrenden und häufigen Gefährdung in Deutschland weiter und zunehmend im Fokus bleiben.

### Supplementary Information

Die zusätzlichen Onlinematerialien (Tab. A1–A9) enthalten weitergehende Informationen zur Stichprobe und zu Gruppenunterschieden (Varianzanalysen).
