# Supplementary material for: Heat and heavy rain prevention measures in daycare centres and care facilities: an evaluation of risk perception, communication and information materials
Source: Bundesgesundheitsblatt Gesundheitsforschung Gesundheitsschutz. 2024 Apr 24;67(6):730–9. [Article in German] doi: 10.1007/s00103-024-03876-8 (PMC11166820; doi:10.1007/s00103-024-03876-8)
Supplement: Supplementary file 1 — Die zusätzlichen Onlinematerialien (Tab. A1–A9) enthalten weitergehende Informationen zur Stichprobe und zu Gruppenunterschieden (Varianzanalysen). [file 103_2024_3876_MOESM1_ESM.pdf]

Zusätzliche Onlinematerialien

Heidenreich, Deppermann, Thieken & Otto (2024): Maßnahmen zur Hitze- und Starkregenvorsorge für Kitas und Pflegeeinrichtungen: Eine Evaluation von Risikowahrnehmung und Informationsmaterialien

Tabelle A1. Übersicht der kontaktierten und erreichten Teilnehmenden.

	**Angeschrieben**	**Teilgenommen**	**Rücklaufquote in %[1]**
Stadtverwaltung	1232	198	16,1
Träger	575	100	17,4
Einrichtungen[2]	-	35	-
Insgesamt	1807	333	18,4

Anmerkung: [1] Die angegebene Rücklaufquote gibt nur eine grobe Tendenz wieder, da zu beachten ist, dass die Befragung zusätzlich über die Webseite beworben wurde und der Einladungslink von einigen Empänger:innen an andere weitergeleitet wurde. [2]Einrichtungen wurden 2021 nicht kontaktiert, daher lässt sich hier keine prozentuale Rücklaufquote berechnen.

Tabelle A2. Abteilung der befragten Mitarbeitenden der Stadtverwaltungen.

	n	%
Gesundheit und Soziales	171	86,4
Umwelt und Klima	17	8,6
Anderes	8	4,0
nicht beantwortet	3	1,5
Gesamt	198	100,0

Anmerkung: Eine Person gab an, im Bereich Umwelt und Gesundheit zu arbeiten, und wurde daher sowohl dem Bereich Gesundheit und Soziales als auch Umwelt und Klima zugeordnet.

Tabelle A3. Überblick Soziodemographie nach Befragtengruppe (Alter, Geschlecht und Bundesland; M: Mittelwert, SD: Standardabweichung, N: Fallzahl).

	**Stadt**		**Träger**		**Einrichtung**		**Gesamt**	
	M	*SD*	*M*	*SD*	*M*	*SD*	*M*	*SD*
**Alter**	46,9	11,1	48,8	9,6	50,8	9,5	47,9	10,5
N	152		79		29		260	

**Geschlecht**	*N*	*%*	*N*	*%*	*N*	*%*	*N*	*%*
weiblich	139	70,2	81	81,0	32	91,4	252	75,7
männlich	36	18,2	15	15,0	1	2,9	52	15,6
divers	1	0,5	1	1,0	0	0,0	2	0,6
nicht beantwortet	12	6,1	2	2,0	0	0,0	14	4,2
keine Angabe	10	5,1	1	1,0	2	5,7	13	3,9

**Bundesland**	*N*	*%*	*N*	*%*	*N*	*%*	*N*	*%*
Baden-Württemberg	20	10,1	14	14,0	5	14,3	39	11,7
Bayern	34	17,2	6	6,1	3	8,6	43	12,9
Berlin	7	3,5	3	3,0	0	0,0	10	3,0
Brandenburg	2	1,0	1	1,0	0	0,0	3	0,9
Bremen	4	2,0	1	1,0	0	0,0	5	1,5
Hamburg	3	1,5	1	1,0	0	0,0	4	1,2
Hessen	11	5,6	4	4,0	3	8,6	18	5,4
Mecklenburg-Vorpommern	0	0,0	3	3,0	0	0,0	3	0,9
Niedersachsen	11	5,6	20	20,0	3	8,6	34	10,2
Nordrhein-Westfalen	31	15,7	15	15,0	2	5,7	48	14,4
Rheinland-Pfalz	8	4,0	24	24,0	7	20,0	39	11,7
Saarland	2	1,0	1	1,0	0	0,0	3	0,9
Sachsen	4	2,0	3	3,0	1	2,9	8	2,4
Sachsen-Anhalt	8	4,0	3	3,0	1	2,9	12	3,6
Schleswig-Holstein	13	6,6	0	0,0	3	8,6	16	4,8
Thüringen	33	16,7	0	0,0	7	20,0	40	12,0
Keine Angabe	7	3,5	1	1,0	0	0,0	8	2,4
**Gesamt**	198	100	100	100	35	100	333	100

Anmerkung: Bei der Frage nach dem Geschlecht war die Auswahl zwischen „weiblich“, „männlich“, „divers“ und „keine Angabe“ möglich. Zusätzlich haben einige Personen die Frage übersprungen, was sich in der Zeile „nicht beantwortet“ widerspiegelt.

Tabelle A4. Ergebnisse einer Varianzanalyse mit Messwiederholung zwischen den Naturgefahren (Hitze/Starkregen als Innersubjektfaktor) und den befragten Gruppen (Zwischensubjektfaktor) hinsichtlich der Risikowahrnehmung (Betroffenheit, Thematisierung, Handlungswissen).

	Hitze		Starkregen				ANOVA		
	M	*SD*	*M*	*SD*	*n*	Effekt	*F*	*df*	η²
Betroffenheit									
Stadtverwaltung	4,1	1,2	2,6	1,3	156	Naturgefahr	171,454	1; 266	0,392**
Träger	4,0	1,3	2,2	1,3	84	Gruppe	2,372	2; 266	0,018
Einrichtung	4,3	1,4	2,6	1,3	29	Interaktion	1,014	2; 266	0,008
Gesamt	4,1	1,3	2,5	1,3	269				
Thematisierung									
Stadtverwaltung	3,8	1,6	2,3	1,3	126	Naturgefahr	114,795	1; 228	0,335**
Träger	3,5	1,6	1,7	1,1	80	Gruppe	3,571	2; 228	0,030
Einrichtung	3,7	1,6	2,0	1,5	25	Interaktion	1,003	2; 228	0,009
Gesamt	3,7	1,6	2,1	1,3	231				
Handlungswissen									
Stadtverwaltung	4,1	1,4	3,1	1,5	122	Naturgefahr	168,279	1; 223	0,430**
Träger	4,4	1,4	2,6	1,6	74	Gruppe	0,463	2; 223	0,004
Einrichtung	4,7	1,1	2,8	1,7	30	Interaktion	8,346	2; 223	0,070**
Gesamt	4,3	1,4	2,9	1,6	226				

Anmerkung: *$p \leq 0{,}01$; **$p \leq 0{,}001$. Unter „Naturgefahr“ wurde zwischen Hitze und Starkregen unterschieden. Als Gruppen wurden jeweils die Stadtverwaltungen, Träger und Einrichtungen verglichen.

Tabelle A5. Ergebnisse einer zweifaktoriellen Varianzanalyse (ANOVA) zwischen den Naturgefahren (Hitze/Starkregen als Innersubjektfaktor) und den befragten Gruppen (Zwischensubjektfaktor) hinsichtlich der Handlungsabsichten (Unterstützung, Empfehlung)

	Hitze		Starkregen				ANOVA		
	M	*SD*	*M*	*SD*	*n*	Effekt	*F*	*df*	η²
Unterstützung									
Stadtverwaltung	3,1	1,3	2,3	1,2	189	Naturgefahr	145,696	1; 280	0,342**
Träger	3,1	1,3	2,2	1,2	93	Gruppe	0,847	1; 280	0,005
Gesamt	3,1	1,3	2,3	1,2	282	Interaktion	0,310	1; 280	0,001
Empfehlung									
Stadtverwaltung	3,4	1,4	2,4	1,3	190	Naturgefahr	163,142	1; 283	0,366**
Träger	3,4	1,3	2,3	1,2	95	Gruppe	0,344	1; 283	0,001
Gesamt	3,4	1,3	2,4	1,3	285	Interaktion	0,866	1; 283	0,003

Anmerkung: *$p \leq 0{,}01$; **$p \leq 0{,}001$. Unter „Naturgefahr“ wurde zwischen Hitze und Starkregen unterschieden. Als Gruppen wurden jeweils die Stadtverwaltungen, Träger und Einrichtungen verglichen.

Unterstützung und Empfehlung wurden mit den folgenden Items erfasst, die auf einer Skala von 1 = „keine Absicht“ bis 5 = „sehr starke Absicht“ beantwortet wurden:

„Wie sehr beabsichtigen Sie, in den kommenden Wochen...

[Unterstützung] Einrichtungen Unterstützungsmöglichkeiten zur Durchführung von Schutzmaßnahmen gegen Hitzewellen/Starkregen aufzuzeigen?

[Empfehlung] Einrichtungen die Durchführung einer oder mehrerer der vorgestellten Schutzmaßnahmen gegen Hitzewellen/Starkregen zu empfehlen?“

Tabelle A6. Vorhandensein eigener Materialien zum Umgang mit Starkregen und/oder Hitze in den Stadtverwaltungen und bei Trägern

	Stadtverwaltung		Träger	
	N	%	*N*	%
Materialien zum Umgang mit Starkregen vorhanden	25	12,6	6	6,0
Materialien zum Umgang mit Hitze vorhanden	62	31,3	41	41,0
Materialien zum Umgang mit beiden Naturgefahren vorhanden*	20	10,1	5	5,0
Keine Materialien vorhanden	51	25,8	39	39,0
Keine Angabe	3	1,5	2	2,0
Weiß nicht	77	38,9	17	17,0
Gesamt	198		100	

Anmerkung. * Diese Zeile stellt die Schnittmenge aus beiden darüberliegenden Zeilen dar.

Tabelle A7. Kontakt mit Einrichtungen

	Stadtverwaltung		Träger	
	N	%	*N*	%
Wir stehen in Kontakt mit Kitas.	93	47,0	63	63,0
Wir stehen in Kontakt mit Pflegeeinrichtungen.	89	44,9	31	31,0
Wir stehen sowohl mit Kitas als auch Pflegeeinrichtungen in Kontakt. *	31	15,7	6	6,0
Weder noch/keine Angabe	47	23,7	12	12,0
Gesamt	198		100	

Anmerkung. * Diese Zeile stellt die Schnittmenge aus beiden darüberliegenden Zeilen dar.

Tabelle 8. Vorhandensein und Nutzung von Verteilern mit Einrichtungen unter Stadtverwaltungen und Trägern.

	Stadtverwaltung		Träger	
	N	%	*N*	%
Verteiler für Kitas vorhanden	103	52,0	60	60,0
Verteiler für Pflegeeinrichtungen vorhanden	92	46,5	29	29,0
Davon: Verteiler für beide vorhanden	38	19,2	11	11,0
Keine Verteiler Vorhanden	3	1,5	3	3,0
Kanäle zur Kommunikation mit Kitas				
E-Mail	98	49,5	59	59,0
Telefon	36	18,2	19	19,0
Fax	8	4,0	3	3,0
Post	33	16,7	14	14,0
Andere	27	13,6	15	15,0
Kanäle zur Kommunikation mit Pflegeeinrichtungen				
E-Mail	88	44,4	28	28,0
Telefon	27	13,6	8	8,0
Fax	9	4,5	2	2,0
Post	22	11,1	4	4,0
Andere	24	12,1	10	10,0
Kommunizierte Inhalte mit Kitas				
Warnungen	48	24,2	25	25,0
Informationen/Materialien zur Starkregenvorsorge	20	10,1	9	9,0
Informationen/Materialien zur Hitzevorsorge	43	21,7	25	25,0
Neue Beschlüsse, Verordnungen, o.ä.	84	42,4	54	54,0
Kommunizierte Inhalte mit Pflegeeinrichtungen				
Warnungen	47	23,7	16	16,0
Informationen/Materialien zur Starkregenvorsorge	17	8,6	8	8,0
Informationen/Materialien zur Hitzevorsorge	50	25,3	22	22,0
Neue Beschlüsse, Verordnungen, o.ä.	72	36,4	21	21,0
Gesamt	198		100	

Tabelle A9. Ergebnisse der einfaktoriellen Varianzanalysen zum Gruppenunterschied der Wahrnehmung der Handlungsempfehlungen. Zusätzlich angegeben sind Mittelwerte (M), Standardabweichungen (SD) und die jeweilige Anzahl von Angaben je Gruppe (n).

				ANOVA			
	M	*SD*	*n*	Effekt	*F*	*df*	part. η^2
neu vs. bekannt							
Stadtverwaltung	3,55	1,34	195	Gruppe	3,900	2,324	0,024
Träger	3,37	1,49	98				
Einrichtung	2,82	1,59	34				
Gesamt	3,42	1,43	327				
interessant vs. uninteressant							
Stadtverwaltung	1,95	0,99	194	Gruppe	0,619	2,323	0,004
Träger	2,02	1,00	98				
Einrichtung	2,15	1,16	34				
Gesamt	1,99	1,01	326				
hilfreich vs. nutzlos							
Stadtverwaltung	1,93	0,92	194	Gruppe	0,150	2,323	0,001
Träger	1,90	0,88	98				
Einrichtung	2,00	1,18	34				
Gesamt	1,93	0,94	326				
gut anwendbar vs. nicht nutzbar							
Stadtverwaltung	2,20	0,91	194	Gruppe	0,849	2,322	0,005
Träger	2,12	0,88	98				
Einrichtung	2,36	1,11	33				
Gesamt	2,19	0,92	325				
